# Rapid identification of Corynebacterium diphtheriae clonal group associated with diphtheria epidemic, Russian Federation.

**DOI:** 10.3201/eid0701.010119

**Published:** 2001

**Authors:** S Kombarova, C Kim, V Melnikov, M Reeves, O Borisova, I Mazurova, T Popovic

**Affiliations:** Gabrichevsky Institute of Epidemiology and Microbiology, Moscow, Russia.

## Abstract

We used 199 Corynebacterium diphtheriae isolated from 1995 to 1997 in Russia to evaluate the ability of random amplified polymorphic DNA (RAPD) to identify the unique clonal group that emerged there in 1990. Our data show that RAPD can reliably, reproducibly, and rapidly screen a large number of strains to identify the epidemic clonal group.


### Rapid Identification of *Corynebacterium diphtheriae* Clonal Group Associated with Diphtheria Epidemic, Russian Federation

**Svetlana Kombarova,* Chung Kim,† Viatcheslav Melnikov,* Michael Reeves,† Olja Borisova,* Izabella Mazurova,* and Tanja Popovic*†**

*Gabrichevsky Institute of Epidemiology and Microbiology, Moscow, Russia; †Centers for Disease Control and Prevention, Atlanta, Georgia, USA

We used 199 *Corynebacterium diphtheriae* isolated from 1995 to 1997 in Russia to evaluate the ability of random amplified polymorphic DNA (RAPD) to identify the unique clonal group that emerged there in 1990. Our data show that RAPD can reliably, reproducibly, and rapidly screen a large number of strains to identify the epidemic clonal group.

Molecular subtyping, primarily by multilocus enzyme electrophoresis (MEE) and ribotyping, has identified substantial genetic diversity within the *Corynebacterium diphtheriae* species, leading to the identification of a unique clonal group that emerged in Russia in 1990 at the beginning of the current diphtheria epidemic (1). Strains of this clonal group belong to a distinct electrophoretic type complex (ET8 complex) and are of ribotypes G1 and G4. Identification of this clonal group has permitted precise monitoring of the epidemic's growth and rapid detection of imported cases in neighboring and other European countries.

Use of traditional subtyping methods in monitoring the expansion of the epidemic clone has helped differentiate epidemic, endemic, and imported cases and has allowed timely preventive measures. Even as the epidemic declines (from more than 50,000 cases in 1995 to 1,436 cases in 1998), identifying organisms belonging to this epidemic clone in cases of suspected importation into locations where diphtheria is rarely encountered continues to provide valuable information. Since both ribotyping and MEE are time-consuming, taking 3-4 working days to produce results, rapid methods that could distinguish the predominant clone would improve epidemic surveillance and prevention measures.

Random amplified polymorphic DNA (RAPD) is a simple and rapid molecular subtyping method. Recently, Nakao et al. (2) optimized and standardized this assay for *C. diphtheriae* and showed that the discrimination level obtained by RAPD correlated well with that of ribotyping; each of 20-plus ribotyping patterns was associated with one or more distinct RAPD patterns. We compared these two techniques on a large number of *C. diphtheriae* Russian isolates from 1995 to 1997, focusing on the ability of RAPD to identify the isolates of the epidemic G1/4 clonal group.

### The Study

All *C. diphtheriae* strains were collected by the Russian Federal Diphtheria Diagnosis Reference Laboratory. Of 199 isolates from different regions of Russia, 187 were isolated from 1995 to 1997; 12 were isolated during 1993 to 1994; 68 were recovered from clinically diagnosed diphtheria patients; and the remaining 131 isolates were obtained from carriers.

Identification, biotype, and toxigenicity determination were performed by standard

Address for correspondence: Dr. Tanja Popovic, Centers for Disease Control and Prevention, Mail Stop G34, 1600 Clifton Rd., Atlanta, GA 30333, USA; fax: 404-639-3172; e-mail: txp1@cdc.gov.

microbiologic methods (3,4). RAPD was performed by the Ready-To-Go RAPD Kit (Pharmacia Biotech, Piscataway, NJ)(2). RAPD type designations were adopted from those previously documented by Nakao et al. (2). Ribotyping was carried out as previously described (1) with some modifications (Table). MEE was performed as previously described (1). The genetic relatedness of the electrophoretic types (ETs) was illustrated as a dendrogram, generated by the average-linkage method of clustering the ETs (7) and by using an SAS macro program described by Jacobs (8).

Of the 199 *C. diphtheriae* isolates, 185 were biotype gravis, and 14 were biotype mitis. All isolates were toxigenic by the Elek assay. When assayed by RAPD using primers 3 and 4, the 199 isolates that were identical by primer 3 were also identical by primer 4, with the single exception of isolate B506. Of 185 isolates of the gravis biotype, 183 were the G1/4 RAPD type. Isolate B325 had a G4v pattern, and isolate B506 had the G1/4 pattern only by primer 4. When primer 3 was used on this isolate, a different pattern was observed.

The RAPD patterns of the 14 isolates of the mitis biotype were distributed into three groups. The first group included three isolates of the G1/4 RAPD type (B294, B399, B400). The second group included 10 isolates that had RAPD patterns types M1 and M1v. The third group included only one isolate (B306), which had a RAPD pattern completely different from any previously assayed strain.

Seventy-nine isolates were ribotyped. Of the 186 isolates with RAPD G1/4 patterns (183 gravis and 3 mitis), 66 were selected to be ribotyped for their geographic and temporal diversity. In addition, all non-G1/4 isolates were ribotyped. The ribotyping results correlated extremely well with the RAPD data (Table, Figure). With one exception, all isolates of the G1/4 RAPD type also had a G1 or G4 ribotype; 40 had the G1 ribotype, and 26 isolates possessed the G4 ribotype. In addition, the G4v ribotype was observed in the isolate with the G4v RAPD pattern. Isolate B306, which had an RAPD type not previously observed, also had a ribotype that did not resemble any previously established ribotypes. Five M1 and five M1v ribotypes were identified among the 10 M1/1v RAPD type isolates.

Twenty-nine isolates of RAPD types G1/4 and ribotypes G1 or G4 were analyzed by MEE. Among all isolates of this group, seven individual ETs, which clustered at a genetic distance of <0.12, were identified; all ETs were members of the previously defined ET8 complex. Only one to three enzyme differences from the ET8 complex were observed among the individual enzyme types (data not shown). The ET8 complex contains 27 ETs, which are related to each other at a genetic distance of 0.20 and have a maximum of four enzyme differences within the complex.

Table. Random amplified polymorphic DNA (RAPD) assay and ribotyping for 79 Russian *Corynebacterium diphtheriae* isolates[a,b]

| Ribotype | Biotype | Number of strains | RAPD Primer 3 | RAPD Primer 4 |
|---|---|---|---|---|
| G1 | G | 38 | G1/4 | G1/4 |
| | M | 2 | G1/4 | G1/4 |
| G4 | G | 25 | G1/4 | G1/4 |
| | M | 1 | G1/4 | G1/4 |
| | G | 1 | New[c] | G1/4 |
| G4v | G | 1 | G4v | G4v |
| M1 | M | 5 | M1/1v | M1/1v |
| M1v | M | 5 | M1/1v | M1/1v |
| New | M | 1 | New | New |

[a]G, biotype gravis; M, biotype mitis. Cultures were kept lyophilized at room temperature or were stored in defibrinated sheep blood and held at -70°C until needed. Before use, the strains were inoculated onto blood agar plates (trypticase soy agar with 5% sheep blood; Becton Dickinson, Cockeysville, MD) and were incubated at 37°C overnight.
[b]DNA for ribotyping was isolated by the universal isolation procedure (5). Hybridization of restricted DNA fragments was performed using a mixture of five digoxigenin-labeled oligonucleotide probes at 37°C for 4 hours as recently described by Regnault et al. (6). Posthybridization washes were also performed at 37°C in 2X SSC (1 X SSC is 0.15 M NaCl plus 0.015 M sodium citrate), 0.1% sodium dodecyl sulfate (SDS) for 2x5 minutes and in 0.1X SSC, 0.1% SDS for 2x10 minutes. Detection was performed by using the DIG Wash and Block Buffer Set (Boehringer Mannheim Biochemicals, Indianapolis, IN), sheep anti-digoxigenin antibody conjugated with alkaline phosphatase, nitroblue tetrazolium chloride (NBT), and 5-bromo-4-chloro-3-indolylphosphate (BCIP).
[c]New = pattern had not been previously observed.

### Conclusions

The *C. diphtheriae* epidemic clonal group associated with the recent diphtheria epidemic in the Russian Federation is characterized as being of ribotypes G1 and G4 and belonging to the ET8 complex. Detection of a unique epidemic clonal group has allowed continuous monitoring of the circulation of existing clones and rapid detection of new or unusual clones. The epidemic emphasized the need for continuous study of the

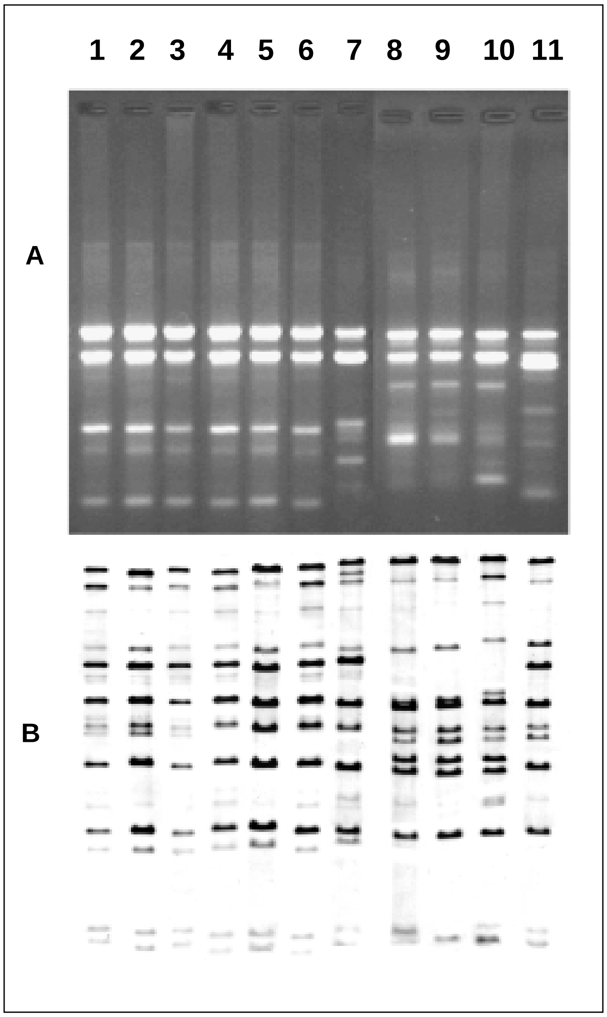

Figure. A) RAPD patterns of *Corynebacterium diphtheriae* isolated from 1995 to 1997. Lane 1, 1740 (strain #), gravis, G1/4 RAPD type strain. Lane 2, B327, gravis, G1/4 (RAPD type), 1997 (year of isolation). Lane 3, B400, mitis, G1/4, 1995. Lane 4, 490, gravis, G1/4 ribotyping type strain. Lane 5, B375, gravis, G1/4, 1995. Lane 6, B294, mitis, G1/4, 1996. Lane 7, B325, gravis, G4v, 1997. Lane 8, 860, mitis, M1/M1v RAPD type strain. Lane 9, B389, mitis, M1/M1v, 1995. Lane 10, B324, mitis, M1/M1v, 1997. Lane 11, B306, mitis, new RAPD pattern, 1997. B) Ribotyping patterns of *C. diphtheriae* isolated from 1995 to 1997. Lane 1, G4174 (strain #), gravis, G1 ribotyping type strain. Lane 2, B327, gravis, G1 (ribotype) 1997 (year of isolation). Lane 3, B400, mitis, G1, 1995. Lane 4, G4183, gravis, G4 ribotyping type strain. Lane 5, B375, gravis, G4, 1995. Lane 6, B294, mitis, G4, 1996. Lane 7, B325, gravis, G4v, 1997. Lane 8, G4212, mitis, M1 ribotyping type strain. Lane 9, B389, mitis, M1, 1995. Lane 10, B324, mitis, M1v, 1997. Lane 11, B306, mitis, new ribotype, 1997.

biologic properties of *C. diphtheriae*. Thus, a *Corynebacterium* ribotype database has been established, and substantial efforts are under way to standardize molecular subtyping approaches in diphtheria reference centers worldwide (9).

Given that ribotyping still takes several days to be completed, we evaluated the role RAPD might have as a rapid and reliable molecular subtyping tool by comparing its differentiation abilities to those of ribotyping; 199 *C. diphtheriae* isolates were analyzed, and RAPD was shown to be as discriminative as standard ribotyping. All but one isolate of ribotypes G1 or G4 were correctly identified as belonging to the G1/4 RAPD group by both primers. For comparative purposes, we analyzed a smaller number of isolates with M1 and M1v ribotypes; all of these isolates were also correctly identified as belonging to the M1/1v group by both RAPD primers. The two isolates that gave non-G1/4 or M1/1v ribotypes (B325 and B306) were obtained in the Asian part of Russia (Barnaul and Cheljabinsk, respectively).

Furthermore, of the 29 isolates that were analyzed by MEE, 7 closely related ETs (all members of the ET8 complex) were identified. These ETs differed from the predominant ET8 by one to three enzymes. MEE still provides a higher level of differentiation of the epidemic *C. diphtheriae* isolates (27 ETs) than ribotyping and RAPD (2 and 1 types, respectively). However, by all three methods, the isolates in our study were still clearly defined as belonging to the earlier described epidemic clonal group.

Our data unambiguously show that RAPD can be reliably and reproducibly used for rapidly screening strains of the predominant epidemic clonal group. Such rapid identification is extremely useful in investigations of potentially imported cases so that timely preventive measures can be implemented.

Dr. Kombarova is a microbiologist at the Gabrichevsky Institute of Epidemiology and Microbiology in Moscow, Russia. Her areas of expertise are isolation, identification, and molecular subtyping of *Corynebacterium diphtheriae*. She was extensively involved in identifying the epidemic clonal group associated with the diphtheria epidemic in Russia.

### References

1. Popovic T, Kombarova S, Reeves M, Nakao H, Mazurova IK, Wharton M, et al. Molecular epidemiology of diphtheria in Russia, 1985-1994. J Infect Dis 1996;174:1064-72.
2. Nakao H, Popovic T. Use of random amplified polymorphic DNA for rapid molecular subtyping of *Corynebacterium diphtheriae*. Diagn Microbiol Infect Dis 1998;30:167-72.
3. Efstratiou A, Maple PA. WHO manual for the laboratory diagnosis of diphtheria. Geneva: World Health Organization; 1994. Reference no. ICP-EPI 038(C).
4. Mazurova IK, Melnikov V, Kombarova S. Manual for laboratory diagnostis of diphtheria infection. Moscow: Russian Federation State Committee on Sanitary Epidemiologic Surveillance;1995.
5. Graves LM, Swaminathan B. Universal bacterial DNA isolation procedure. In: Persing DH, Smith TR, Tenover FC, White TJ, editors. Diagnostic molecular microbiology. Washington: American Society for Microbiology; 1993. p. 617-21.
6. Regnault B, Grimont R, Grimont PAD. Universal ribotyping method using a chemically labeled oligonucleotide probe mixture. Res Microbiol 1997;146:649-59.
7. Selander RK, Daugant DA, Ochman H, Musser JM, Gilmore MN, Whittam TS. Methods of multilocus enzyme electrophoresis for bacterial population genetic and systematics. Appl Environ Microbiol 1986;51:873-4.
8. Jacobs D. SAS/GRAPH software and numerical taxonomy. In: Proceedings of the 15th Annual Users Group International Conference. Cary (NC): SAS Institute; 1990. p. 1413-18.
9. Grimont PAD, Grimont F, Ruckly C. The *Corynebacterium diphtheriae* ribotype database. In: Program and abstracts of the 5th International Meeting of the European Laboratory Working Group on Diphtheria. London: Public Health Laboratory Service; 1998. p. 31.
